# Hemolysis and Pulmonary Insufficiency following Right Ventricular Assist Device Implantation

**DOI:** 10.1155/2012/376384

**Published:** 2012-09-26

**Authors:** Sarah A. Schubert, Behzad Soleimani, Walter E. Pae

**Affiliations:** Department of Cardiothoracic Surgery, Penn State Hershey Heart and Vascular Institute, 500 University Drive, Hershey, PA 17033, USA

## Abstract

We report a case of severe hemolysis and pulmonary valve insufficiency (PI) following right ventricular support using a paracorporeal pneumatic pump (Abiomed, Danvers, MA, USA). We speculate that the high velocity jet of blood emanating from the outflow cannula caused turbulence above the pulmonary valve, leading to PI and hemolysis. Despite the growing number of implanted ventricular assist devices, we could find no report in the literature describing pulmonary valve insufficiency secondary to right ventricular assist device (RVAD) placement. Fortunately, in this case, right ventricular function recovered sufficiently after seven days of support, allowing explantation of the device and resolution of PI and hemolysis.

## 1. Introduction

Although cardiac transplantation remains the gold standard for the treatment of end-stage heart failure, the demand for transplantable hearts exceeds the supply. Fortunately, ventricular assist devices have emerged as a viable bridging therapy until a donor heart is obtained. Despite their uncomplicated design and relatively straightforward placement in the chest, care must be taken during pump and cannulae positioning, and patients must be monitored closely following surgery. Here we present a case of hemolysis and PI following right ventricular support with a biventricular assist device.

A 21-year-old Hispanic female was diagnosed with peripartum cardiomyopathy at 30 weeks' gestation. She became refractorily inotrope-dependent with a cardiac index of 1.4, pulmonary vascular resistance of 3.5 to 4.5 Wood units, right atrial pressure of 24 mmHg, and pulmonary capillary wedge pressure of 22 mmHg, eventually developing cardiogenic shock and multiorgan dysfunction. Because of continued low output, she received an Abiomed 5000 paracorporeal biventricular pneumatic assist device (BiVAD) and was listed as a status 1A transplant candidate.

**Figure 1:**
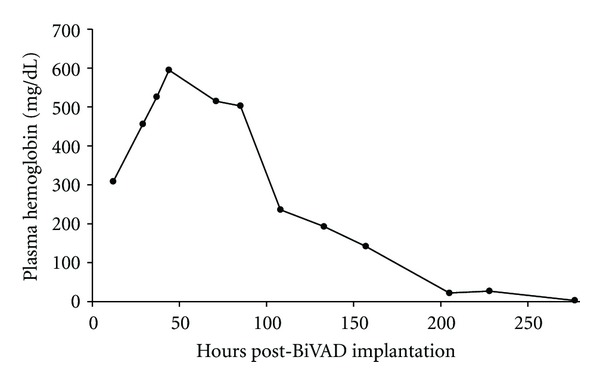
Plasma hemoglobin (mg/dL) following biventricular assist device (BiVAD) implantation.

**Figure 2:**
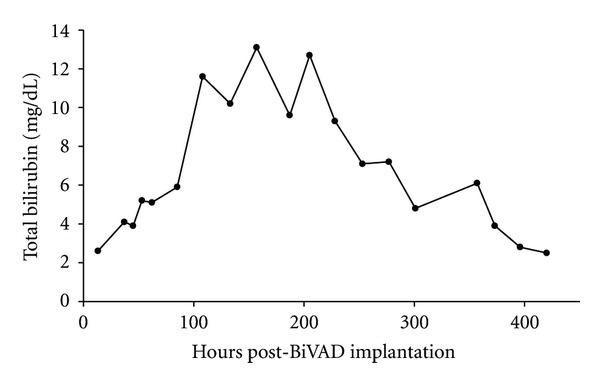
Total bilirubin (mg/dL) following biventricular assist device (BiVAD) implantation.

Following BiVAD implantation, the patient developed severe hemolysis, with plasma hemoglobin levels peaking at 595 mg/dL (Figure 1) on the third postoperative day and a total bilirubin reaching 13.1 mg/dL (Figure 2). 

**Figure 3:**
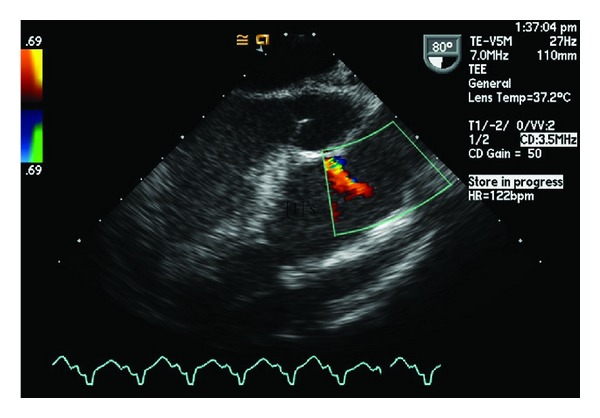
Transesophageal echo demonstrating the pulmonary insufficiency was believed to be secondary to the position of the outflow cannula of the right ventricular assist device.

Transesophageal echocardiogram (TEE) demonstrated turbulence above the pulmonic valve and massive PI that correlated with RVAD flow rate (Figure 3).

In order to decrease shear stresses, both pumps were returned to right-sided driving pressures with minimal vacuum. Hemolysis continued following these adjustments—albeit to a lesser extent—with plasma hemoglobin levels of approximately 200 mg/dL. 

We believed the PI to be secondary to the jet from the outflow cannula of the RVAD on the pulmonic valve and also likely responsible for the high shear stresses and hemolysis. When the rate of the right ventricular pump was decreased, TEE showed improvement in right ventricular function (as compared to that intraoperatively), with maintenance of central venous pressure, near normal pulmonary artery pressures, and excellent left-sided flows. With the improvement in right heart function seen by the seventh postoperative day and continuing RVAD-induced hemolysis, she was accordingly taken to the operating room for removal of the RVAD.

The patient tolerated the RVAD explant well, and a postoperative TEE showed no residual PI. Additionally, markers of hemolysis continued to decline. After reoperation for tamponade following explant, the left heart remained device-dependent, but right heart function was equivocal, requiring significant inotropic support. At this point, a donor heart became available, and 14 days after the BiVAD implantation, the patient underwent an uneventful orthotopic cardiac transplantation and removal of the LVAD. During the recipient cardiectomy, the native pulmonary valve was found to be structurally normal on inspection. The patient had an uncomplicated recovery and was discharged home on the tenth postoperative day.

## 2. Discussion

 Cardiac transplantation offers the greatest morbidity and mortality benefit for patients experiencing end-stage heart failure; however, the scarcity of donor hearts limits its viability as a treatment option. Yet, with continuing developments in mechanical circulatory support technology, ventricular assist devices are becoming more reliable and patient outcomes with such devices are improving. The VAD can be used as a bridge to recovery, a bridge to transplantation until a donor heart is obtained, or as destination therapy for those patients who are not transplant candidates [1]. In those patients who qualify for cardiac transplantation, the primary indications for VAD implantation as bridge-to-transplant therapy are refractory arrhythmia or worsening hemodynamics, in spite of maximal inotropic support [2].

 The ultimate clinical goals of VAD therapy are to restore adequate blood flow, preserve end-organ function, and provide effective decompression of the failing ventricle(s). With a relatively unsophisticated design, VADs augment ventricular function, hence supporting systemic perfusion—by mechanically pumping blood from the RV to the pulmonary circulation with an RVAD or from the LV to the systemic circulation through the aorta with an LVAD. The inflow cannula of the LVAD—usually placed at the apex of the heart—directs blood from the left atrium or ventricle into the pump. The outflow cannula is anastomosed to the ascending aorta where blood can then reach the systemic circulation. In the RVAD, the inflow cannula directs blood from the right atrium or ventricle to the pump, and the outflow cannula is anastomosed to the main pulmonary artery [3].

 In this case, the low position of the RVAD outflow cannula (chosen to facilitate later transplantation) produced a jet of blood such that the velocity across the pulmonic valve was increased. The calculated Reynolds number is increased behind the pulmonary valve because of the mixing of blood flowing from the right ventricle and the RVAD, thus accounting for the pulmonary insufficiency and clinical significant hemolysis. As the output from the RVAD was decreased, the pulmonary regurgitation also decreased, further suggesting that the velocity of the jet from the RVAD was directly responsible for the increased turbulence behind the pulmonic valve [4].

Additionally, the increased turbulence decreases the pressure before the pulmonic valve in the right ventricle. In a classic Bernoulli effect, this widened pressure gradient across the pulmonic valve increases the velocity of the regurgitated blood through the pulmonic valve, resulting in greater PI [5]. This case represents the only report we could find in the literature detailing significant PI following appropriate RVAD placement.

Despite the relatively straightforward placement of these devices, this case demonstrates the necessity of monitoring flow dynamics within the pump and the heart following VAD implantation to appropriately address relevant anatomical and functional factors. This same mechanism may account for the aortic regurgitation now being seen with continuous flow devices.
